# The ratio of total cholesterol to high density lipoprotein cholesterol and myocardial infarction in Women’s health in the Lund area (WHILA): a 17-year follow-up cohort study

**DOI:** 10.1186/s12872-019-1228-7

**Published:** 2019-10-29

**Authors:** Susanna Calling, Sven-Erik Johansson, Moa Wolff, Jan Sundquist, Kristina Sundquist

**Affiliations:** 1 Center for Primary Health Care Research, Skåne University Hospital Lund, Lund, Sweden; 2 Department of Clinical Sciences Malmö, Lund University, Lund, Sweden; 3 Clinical Research Centre, Box 50332, 202 13 Malmö, Sweden

**Keywords:** Hyperlipidemias, Lipoproteins, Cholesterol, HDL, Myocardial infarction, Cardiovascular diseases, women

## Abstract

**Background:**

Identifying variables predictive of acute myocardial infarction (AMI) in women is important. The use of the ratio of total cholesterol-to-high density lipoprotein cholesterol (TC/HDL-C) is often overlooked. The aim was to study TC/HDL-C in relation to later AMI, in a large sample of women, adjusted for age, educational status, smoking, waist-hip ratio, blood pressure, and neighbourhood socioeconomic status. The hypothesis was that increasing TC/HDL-C is associated with an increased risk of later AMI.

**Methods:**

From December 1995 to February 2000, 6147 women aged 50–59 years from the Womens’ Health in Lund area (WHILA) study in southern Sweden underwent a physical examination, laboratory tests and filled in a questionnaire. The women were followed through national registers for incidence of AMI during a mean follow up of 17 years.

**Results:**

An increasing TC/HDL-C showed a strong relationship with AMI, with the lowest hazard ratio (HR = 1) in women with a ratio of ≤3.5. The HR for AMI was 1.14 (95% CI: 0.73–1.78) for those with a ratio between 3.5 and 4.0; in those with a ratio between 4.0 and 5.0 the HR for AMI was 1.46 (95% CI: 1.00–2.13) and in those with a ratio > 5.0 the HR was 1.89 (95% CI 1.26–2.82), after adjusting for potential confounding factors.

**Conclusions:**

TC/HDL-C ratio is a powerful predictor of AMI in middle-aged women. The results indicate that this variable should be used in clinical practice and is important for early identification of individuals at risk of AMI.

## Background

Primary prevention and risk prediction are important to reduce the rates of ischemic heart disease (IHD), which is still the leading cause of death globally [1, 2]. Acute myocardial infarction (AMI) is a common consequence of IHD, which occurs when the coronary blood flow decreases or stops to a part of the heart which causes tissue damage and is a major reason to mortality from IHD [3]. In general, the risk for AMI is higher in men than in women [4]. However, heart disease mortality in men accelerates at a relatively young age, whereas in women the risk shows a steep increase later in life, around 60 years of age [5]. Moreover, a recent increase in AMI has been reported among younger women in some countries, e.g. the USA, which may be caused by the obesity epidemic the last decades [1, 3, 6]. Most knowledge on the prevention, diagnosis and treatment of AMI is still based on studies conducted predominantly on men [7], even though it is known that predictive risk factors differ between men and women [8, 9]. AMI in women often presents with diffuse symptoms and remains undertreated in comparison with men [3, 9, 10]. Therefore, it is of high importance for clinicians to identify women with a high risk for AMI, especially in women in their mid-life years before they develop severe coronary atherosclerosis [5, 11, 12].

Hypercholesterolemia is an established cardiovascular risk factor and includes several circulating lipoproteins in the blood. High total cholesterol (TC) and low-density lipoprotein cholesterol (LDL-C) increase the risk of AMI [13], and high-density lipoprotein cholesterol (HDL-C) is a strong protective factor [4, 14]. Earlier studies have suggested a gender heterogeneity; the association between TC and AMI is stronger in men [14–16] and HDL-C seems to be more important in women [12]. Moreover, the ratio of TC to HDL-C (TC/HDL-C) has been suggested as a strong independent predictor for AMI in men, however the clinical use of the ratio is often overlooked [17–20]. Only a few studies have focused on this association in women [21, 22], suggesting that women have lower TC/HDL-C, because of higher HDL-C levels [17, 23]. Few studies have specifically analysed the association between TC/HDL-C in middle-aged women.

In primary health care, different risk prediction equations are used to estimate patients’ future risk of cardiovascular mortality, for example the European Heart SCORE [21]. The SCORE project concluded in 2003, that TC/HDL-C has no advantage over TC alone as a single index of lipid level. However, the recently published PREDICT study used TC/HDL-C ratio in a cardiovascular risk prediction equation derived from 400,000 primary care patients in New Zealand [24]. The PREDICT study concluded that older risk prediction tools need to be recalibrated as they are based on old cohorts and tend to overestimate cardiovascular mortality risk, as the modern population is generally healthier.

In conclusion, AMI is understudied and undertreated in women and seems to be a growing problem in some Western countries, and TC/HDL-C has not been sufficiently studied in middle-aged women [3, 9, 10]. The present study will make a novel contribution to the research field by studying the association between TC/HDL-C and AMI during a 17 year long follow-up, in a large sample of middle-aged women, adjusted for a comprehensive set of potential confounders, i.e., age, educational status, smoking, waist-hip ratio, blood pressure, and neighbourhood socioeconomic status. The hypothesis was that increasing TC/HDL-C is associated with an increasing risk of AMI.

## Methods

The Women’s Health in Lund Area (WHILA) study is a prospective cohort study that invited all women 50–59 years (born between 1935 and 1945) from five southern municipalities in Sweden, to a health survey, and the procedures have been described previously [10, 25]. In short, 6916 women underwent a baseline examination between Dec 1995 and Feb 2000. Due to missing variables, mainly for geographical neighbourhood area codes, a sample of 6147 women was included in the present study. After written consent, the women underwent a physical examination with measurement of body weight, height, minimal waist and maximal hip circumference. Blood pressure (mm Hg) was recorded in the right arm after 15 and 20 min rest in sitting position, and the average of the two measurements was used. Serum levels of TC and HDL-C were measured with a Cholestech LDX-instrument (Cholestech Corporation, Hayward, CA, USA) on capillary whole blood without previous fasting [25, 26]. Furthermore, the participants filled in a questionnaire, including 104 questions about medical history, drug treatments, lifestyle, sociodemographic data and various health problems. If they had any questions, they could ask a nurse. No financial reimbursement was given for participation [10, 25].

### Follow up and outcome variable

The follow up of AMI has been described previously [10]. All included women were followed from the day of the baseline examination, until first hospitalization of AMI, or until the end of the study May 31st 2015, by linking the data to the Hospital Discharge Register. The mean follow-up time was 17 years.

*Acute myocardial infarction (AMI)* was based on a diagnosis documented in the Hospital Discharge Register according to the International Classification of Diseases, i.e. code I21.0-I21.9 (ICD-10) or code 410 (ICD-8). Those who had had an AMI before screening were excluded. Only AMI was included, i.e. other codes of ischaemic heart disease, e.g. angina pectoris, were not included.

### Predictor variable

*Ratio of total cholesterol (mmol/L) to HDL-C (mmol/L), TC/HDL-C* was treated as a continuous variable, linearly related to risk of AMI. We also categorized the ratio into four levels, based on clustering of the hazard ratios: (1) ≤3.5; (2) > 3.5 - ≤4.0; (3) > 4.0 - ≤5.0; and (4) > 5.0.

### Explanatory variables

The explanatory variables were based on the examinations and questionnaires at baseline, and have been described previously [10, 25].

*Agec,* age at screening, was treated as a continuous variable, centered around its mean (56 years).

*Education;* Educational level was categorized into low/middle (≤12 years) and high (university).

*Waist hip ratio (WHR)* was calculated as waist circumference (cm) divided by hip circumference (cm) and categorized into two categories ≤0.78 and > 0.78. WHR has been shown to be a significant predictor of AMI [27, 28]. As the sample size was quite small and the included women lived in areas with higher socioeconomic status than the general Swedish population, the cut-off point recommended by Word Health Organization (0.85) could not be used. Therefore, we chose a cut-off of 0.78 as the optimal limit for this sample.

*Blood pressure* was categorized into three levels, based on the distribution: 1) systolic blood pressure < 140 mmHg and diastolic blood pressure < 90 mmHg, 2) systolic blood pressure 140–149 mmHg or diastolic blood pressure 90–99 mmHg, and 3) systolic blood pressure ≥ 150 mmHg or diastolic blood pressure ≥ 100 mmHg. The reason for this categorization instead of the grades of hypertension used by international guidelines, was the small sample size [10, 29, 30].

*Smoking* was categorized into (1) non-smoker (2) former smoker and (3) daily smoker.

*Neighborhood Deprivation Index (NDI)* was used as a proxy for socioeconomic status of the neighbourhood area, and has previously been described in detail [31]. The higher the NDI, the more deprived the neighbourhood. In the present study, NDI was measured in 1995 and dichotomized into affluent (0) and deprived (1) areas. The cut point of the z-score of NDI was set to − 1.1. As there was a relatively high number of missing values for NDI (e.g. individuals who missed a geographical code), which decreased the sample to 6147 women. In the sample, 81% of the women lived in areas with NDI < 0 (z-score). In the statistical analyses, the variable was treated as an individual variable, as the small sample size was not suitable for multi-level analysis.

We found no associations between AMI and postmenopausal hormone therapy use, age at menopause, family history of cardiovascular disease, physical activity or self-reported diabetes. Thus, none of these variables was included.

### Statistical method

Some of the statistical procedures have been described previously [10]. The data was weighted by age and county so that the missing data was compensated: N_i_/n_i_ responders per one-year age-group (50–59) and municipality. There was a variation in response rate in the different age-groups between 58.9 (youngest) and 66.7% (oldest), in average 64.2%. The weights sum up to population size 1995.

The incidence rate of AMI (formed from the number of failures divided by the person-time, per 10,000 person years at risk) was estimated with 95% confidence intervals (STPTIME in STATA) per variable.

A Cox regression model was used to analyse the association between the ratio TC/HDL-C and AMI, with adjustment for all the potential confounders, i.e. agec, education, WHR, blood pressure, smoking and NDI. All included variables satisfied the proportional hazard assumption. There were no interactions between the ratio TC/HDL-C and any of the other included covariates. The continuous relationship (Hazard ratios (HR) with 95% confidence interval (CI)) between the ratio TC/HDL-C and AMI was estimated by restricted cubic splines in two models, one model adjusted for age and another adjusted for all included variables.

STATA version 13 was used for the statistical analyses.

### Non-response analyses

Comparing the distribution of the different variables among responders and non-responders (those who only responded partially), we found significances in education (fewer with low education among non-responders) and in TC/HDL-C (fewer with high ratios among non-responders). However, responders and non-responders had equal risk of AMI in a Cox regression model adjusted for age. We conclude that non-responders should only influence the results marginally.

## Results

The distributions of the characteristics of the women are shown by the categorized variable of TC/HDL-C (Table 1). The women were on average 56 years of age at the screening in all the four categories of TC/HDL-C. Smoking, high WHR, high blood pressure and deprived neighbourhood area were more prevalent with higher category of TC/HDL-C. During the mean follow-up period of 17 years, 191 women (3.1%) suffered AMI. The incidence rates (IR) for AMI are presented in Table 2.

**Table 1:** Distribution (means and %) of the variables by the ratio total cholesterol and HDL, *n* = 6147

Variable		TC/HDL-C category			
	Totals	≤3.5	> 3.5 - ≤ 4.0	> 4.0 - ≤ 5	> 5.0
Number of women (%)	6147	3326 (54.0)	981 (16.0)	1115 (18.2)	725 (11.9)
Ratio TC/HDL-C (mean)	3.66	2.84	3.73	4.43	6.15
TC (mean; mmol/L)	5.95	5.58	6.07	6.35	6.90
HDL-C (mean; mmol/L)	1.73	1.99	1.63	1.44	1.15
Age, mean (years)	56.4	56.2	56.6	56.7	56.7
Age groups (years)					
50–54 (%)	31.7	34.6	26.8	28.8	29.5
55–59 (%)	49.7	49.1	53.8	49.4	47.5
60–64 (%)	18.6	16.3	19.4	21.8	23.0
Education					
Low-Middle (%)	64.6	62.5	67.0	74.3	65.4
High (%)	35.4	37.5	33.0	25.7	34.6
Smoking					
Non-smoker (%)	59.2	62.8	59.3	55.5	48.2
Former smoker (%)	20.1	20.6	20.9	18.8	19.2
Daily smoker (%)	20.7	16.6	19.8	25.7	32.6
WHR					
Small (≤0.78) (%)	54.7	66.0	53.1	41.0	26.3
Large (> 0.78) (%)	45.3	34.0	46.9	59.0	73.7
Blood pressure					
SBP < 140 & DBP < 90 mmHg (%)	52.2	56.4	53.4	46.4	40.8
SBP 140–149 or DBP 90–99 mmHg (%)	27.1	25.3	25.9	30.5	31.6
SBP ≥ 150 or DBP ≥ 100 mmHg (%)	20.7	18.3	20.7	23.1	27.6
NDI					
Deprived areas (%)	69.8	68.7	68.6	71.7	73.3
Affluent areas (%)	30.2	31.3	31.4	26.7	26.7

*TC* Total cholesterol, *HDL* High-density-lipoprotein cholesterol, *TC/HDL-C* Total-cholesterol-to-HDL ratio, *WHR* Waist-hip-ratio, *SBP* Systolic blood pressure, *DBP* Diastolic blood pressure, *NDI* Neighborhood deprivation index

**Table 2:** Incidence rates (IR) per 10,000 person-years at risk and unadjusted hazard ratios (HR). *n* = 6147; AMI = 191

Variable	AMI	Incidence rates		Unadjusted HR
	n	IR	95% CI	HR (95% CI)
Age				
50–54	51	15.3	11.7–20.4	1 (Reference)
55–59	90	17.6	14.4–21.7	1.17 (0.83–1.65)
60–64	50	27.9	21.3–37.3	1.89 (1.21–2.80)
Education				
Low-Middle	136	20.7	17.6–24.6	1.40 (1.02–1.91)
High	55	15.0	11.6–19.7	1 (Reference)
Smoking				
Non-smoker	81	13.4	10.8–16.8	1 (Reference)
Former smoker	39	18.7	13.8–26.1	1.41 (0.96–2.06)
Daily smoker	71	34.1	27.2–43.4	2.58 (1.88–3.56)
WHR				
Small (≤0.78)	78	13.6	10.6–16.4	1 (Reference)
Large (> 0.78)	113	25.0	20.8–30.2	1.87 (1.40–2.50)
Blood pressure (mmHg)				
SBP < 140 & DBP < 90	83	15.4	12.5–19.3	1 (Reference)
SBP 140–149 or DBP 90–99	55	19.9	15.4–26.2	1.30 (0.92–1.83)
SBP ≥ 150 or DBP ≥ 100	53	25.3	19.4–33.5	1.66 (1.17–2.34
TC/HDL-C ratio				
≤ 3.5	74	13.3	10.7–16.9	1 (Reference)
> 3.5 - ≤ 4.0	28	17.0	11.9–25.2	1.28 (0.83–1.98)
> 4.0 - ≤ 5.0	46	24.6	18.6–33.3	1.84 (1.27–2.67)
> 5.0	43	36.3	27.2–49.7	2.75 (1.88–4.02)
NDI				
Affluent areas	40	12.8	9.5–17.8	1 (Reference)
Deprived areas	151	21.2	18.1–25.0	1.65 (1.16–2.34)

*AMI* Acute myocardial infarction, *WHR* Waist-hip-ratio, *SBP* Systolic blood pressure, *DBP* Diastolic blood pressure, *CNI* Care Need Index

**Fig. 1:**
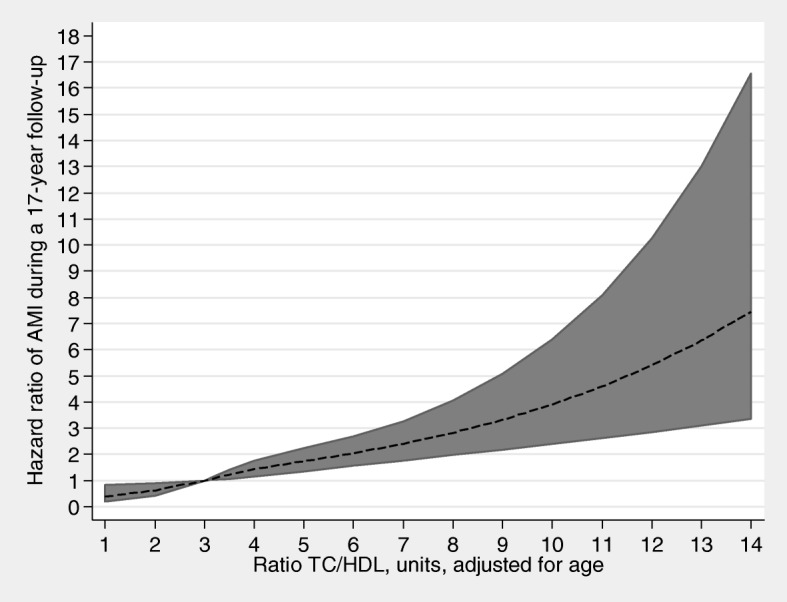
Hazard ratios (HR) with 95% confidence interval (CI) for AMI and TC/HDL-C ratio. Legend: HR estimated by restricted cubic splines. The model is adjusted for age. Those with AMI before screening were excluded. *n* = 6147; AMI = 191

**Fig. 2:**
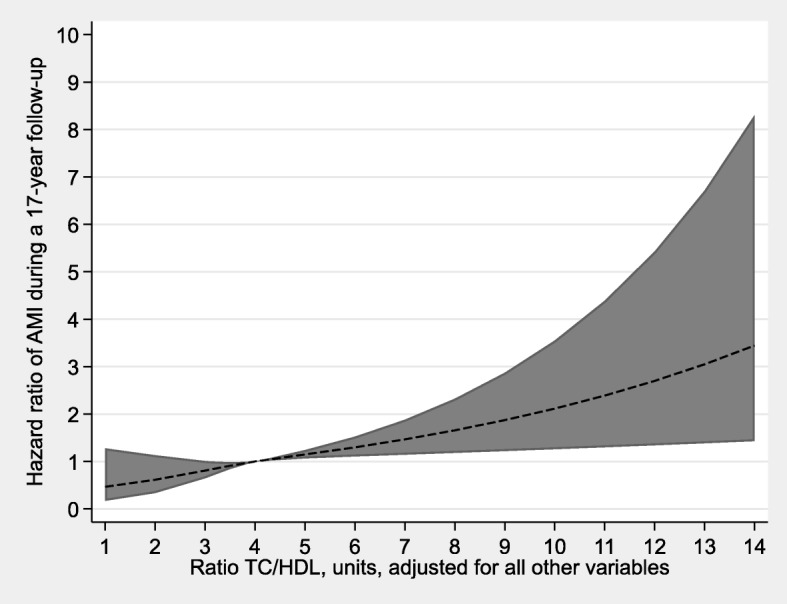
Hazard ratios (HR) with 95% confidence interval (CI) for AMI and TC/HDL-C ratio. Legend: HR estimated by restricted cubic splines. The model is adjusted for all other variables. Those with AMI before screening were excluded. *n* = 6147; AMI = 191

In Table 3, hazard ratios (HR) for AMI are presented, with adjustments for age respectively all included variables. We found a strong association between TC/HDL-C and AMI, HR = 1.16 (95% CI: 1.08–1.25) per 1 unit increase in TC/HDL-C, after adjustments for all included variables. Figures 1 and 2 clearly show a linear association; the higher the TC/HDL-C, the higher the risk of AMI. In a categorized analysis we found: those women with a ratio of ≤3.5 had the lowest hazard ratio (HR = 1) for AMI; those with a ratio between 3.5 and 4.0 had a HR of 1.14 (95% CI: 0.73–1.78); those with a ratio between 4.0 and 5.0 a HR of 1.46 (95% CI: 1.00–2.13); and those with a ratio of > 5.0 a HR of 1.89 (95% CI 1.26–2.82), after adjusting for potential confounding factors, i.e. agec, education, WHR, blood pressure, smoking and NDI (data not shown).

**Table 3:** Hazard ratios (HR) with 95% confidence interval (CI) for AMI, age-adjusted and main effect model. *n* = 6147; AMI = 191

Variable	HR	95% CI
AMI (age-adjusted models)		
Agec (age-56) only age	1.08	1.03–1.13
Education		
Low-Middle	1.31	0.95–1.80
High	1	Ref
Smoking		
Non-smoker	1	Ref
Former smoker	1.43	0.98–2.10
Daily smoker	2.70	1.96–3.71
WHR		
Small (≤ 0.78)	1	Ref
Large (> 0.78)	1.80	1.34–2.42
Blood pressure (mmHg)		
SBP < 140 & DBP < 90	1	Ref
SBP 140–149 or DBP 90–99	1.25	0.89–1.76
SBP ≥ 150 or DBP ≥ 100	1.54	1.08–2.18
TC/HDL-C ratio		
Per 1 unit increase	1.23	1.15–1.31
NDI		
Affluent areas	1	
Deprived areas	1.63	1.15–2.31
AMI (main effects model^a^)		
Agec (age-56)	1.07	1.02–1.13
Education		
Low-Middle	–	–
High	–	–
Smoking		
Non-smoker	1	Ref
Former smoker	1.40	0.96–2.07
Daily smoker	2.47	1.78–3.41
WHR		
Small (≤ 0.78)	1	Ref
Large (> 0.78)	1.45	1.07–1.97
Blood pressure (mmHg)		
SBP < 140 & DBP < 90	1	Ref
SBP 140–149 or DBP 90–99	1.21	0.85–1.71
SBP ≥ 150 or DBP ≥ 100	1.41	0.98–2.03
TC/HDL-C ratio		
Per 1 unit increase	1.16	1.08–1.25
NDI		
Affluent areas	1	
Deprived areas	1.54	1.09–2.18

^a^Adjustments for all included variables

Furthermore, an increased future risk for AMI was associated with age, smoking, large WHR, living in a deprived area and high blood pressure (not statistically significant in the main effect model), Table 3.

## Discussion

In this prospective cohort study of middle-aged women with a mean follow-up of 17 years, we found a strong association between the TC/HDL-C ratio and AMI, after adjustment for age, educational status, smoking, waist-hip ratio, blood pressure and neighbourhood socioeconomic status. The results showed that the higher the TC/HDL-C, the higher the risk of AMI. Other studies have found similar results in men [17–20, 23], however studies on middle-aged women are scarce and don’t show a consistent strong association [21, 22, 32]. For example, one study concluded that TC/HDL-C underestimates the risk for AMI in comparison with apoB/apoA-I ratio [32] and results from the SCORE project concluded that TC/HDL-C is not superior to TC alone [21], even though others have concluded that TC/HDL-C is the most efficient IHD predictor [18, 19, 33]. Moreover, there is no consensus for the recommended level of TC/HDL-C, but a ratio of more than 5 appears to be a strong IHD predictor [23]. Other lipoprotein ratios have been used, for example the LDL/HDL ratio for which the greatest risk is above 5 [18]. In a review of lipoprotein ratios, the magnitude of the increased risk was similar for LDL/HDL ratio and TC/HDL-C ratio > 5 [34].

Women generally suffer from AMI at a later age than men, and it has been suggested that other mechanisms, e.g. plaque erosion and changes in blood clotting, contribute in young ages, rather than unfavorable lipid profiles and coronary obstruction with subsequent plaque rupture, which is especially rare in premenopausal women [12, 35]. Moreover, it is known that estrogen contributes to an antiatherogenic lipid profile by decreasing LDL and increasing HDL-C [12, 35]. However, the results of the present study strengthen the importance to initiate preventive actions focused on blood lipids in middle-aged women. The definition of AMI has changed over time. Nowadays, AMI is divided into ST-elevation myocardial infarction and Non-ST-elevation myocardial infarction, with different patterns regarding course, treatment and prognosis. In the present study, all AMI diagnoses were analysed together, and we do not know whether the TC/HDL-C ratio may affect different types of AMI differently.

According to a Swedish study of the declining rates of coronary heart disease mortality between 1986 and 2002, more than half of the decrease was attributable to reductions in major cardiovascular risk factors, mainly a large decrease in TC [36]. During approximately the same period, dietary fat intake and serum cholesterol levels have been decreasing in many countries [36–39]. Favorable trends of TC/HDL-C ratio have been seen the last decades in many Western countries, Japan and South Korea, according to a recently published article by the NCD Risk Factor Collaboration [40]. However, a more recent Swedish study analysed dietary patterns between 1996 and 2014 and found a recent increase in dietary fat intake [41]. This could be related to the increased interest in the public to eat a low-carb and high-fat diet although it has not been proven that is a healthier choice than other types of diets. The last decades obesity has dramatically increased globally, as a consequence of poor lifestyle habits. In Sweden, mean body mass index (BMI) in women increased from 23.1 to 24.3 between 1980 and 2004, and the increase was especially prominent in middle-aged women and in younger birth cohorts [42]. This indicates that the consequences to obesity, e.g. AMI, may be an increasing problem if we do not manage to identify subgroups for early prevention.

Individuals with high TC/HDL-C can to some extent improve their lipid profile by lifestyle changes, i.e. diet and physical activity. In cases these changes are not sufficient, lipid-lowering medication may be relevant in individuals with high or moderate risk for AMI, also as primary prevention [43, 44]. However, the use of lipid-lowering drugs as primary prevention is a debated question and is not completely defined in primary prevention for women [45, 46]. Current international guidelines emphasize to identify high-risk individuals who would benefit from primary prevention [43].

### Strengths and limitations

The strengths of the present study are the prospective design and the large sample of women drawn from the general population, who were followed for AMI during a long follow-up time. The data has also been adjusted for several potential confounders. Another strength is the design with a clinical baseline examination including blood tests, anthropometric measurements and blood pressure, completed with a thorough self-reported questionnaire including a range of health problems.

The study also has some limitations. Even if we were able to control for several potential confounding factors, it is possible that residual confounding exists, such as inflammatory markers. AMI is a highly complex disorder and different types of confounders may occur in different individuals and change over time, which we could not control for. Lipid levels may differ throughout populations and therefore it may not be possible to generalize our results to other populations. Laboratory methodological differences may yield different results for lipid levels; however, these variations will occur in both individuals with and without AMI. We did not have access to other lipid measures such as apoB/apoA-I ratio, which also needs to be tested in similar cohorts [32]. Furthermore, we had no information about lipid lowering medication; however, in the 1990s statin medication was not recommended in Sweden as primary prevention to people without previous cardiovascular disease, so it is likely that only very few individuals had lipid lowering medication. Because of several missing values on NDI, we had to exclude these individuals. Moreover, self-reported data is limited by the individuals’ will to report, and can be influenced by several factors, including social context and social desirability [47]. The non-responders may be different from the responders and non-responders may have an increased risk of AMI [48]. To some extent, we tried to overcome this limitation by analysing non-responders and found no increased risk of AMI in non-responders. Finally, a problem of using baseline questionnaires in follow-up studies is that the data, e.g. the lipid profile, may change over time. However, the TC/HDL-C ratio may be used as one of several risk factors when estimating the AMI risk in middle-aged women.

## Conclusions

The present study shows that the total cholesterol/HDL-cholesterol ratio is a powerful predictor of AMI in middle-aged women. The results indicate that more commonly clinical use of the ratio may contribute to early identification of individuals at risk of AMI, and support the idea of including the ratio in risk assessment tools for women.

## Data Availability

The data that support the findings of this study are available from the Swedish National Board of Health and Welfare and the Center for Primary Health Care Research, but restrictions apply to the availability of these data, which were used under license for the current study, and so are not publicly available. Data are however available from the authors upon reasonable request and with permission of the Swedish National Board of Health and Welfare and the Center for Primary Health Care Research.

## Abbreviations

AMI: Acute myocardial infarction
BMI: Body mass index
CI: Confidence interval
HDL-C: High-density lipoprotein cholesterol
HR: Hazard ratio
IHD: Ischemic heart disease
IR: Incidence rate
LDL-C: Low-density lipoprotein cholesterol
NDI: Neighborhood deprivation index
TC: Total cholesterol
TC/HDL-C: Total cholesterol to HDL-C ratio
WHILA: Women’s health in Lund area
WHR: Waist hip ratio
